# EP12 Chikungunga arthritis mimicking acute seronegative spondyloarthritis

**DOI:** 10.1093/rap/rkaa052.011

**Published:** 2020-11-03

**Authors:** Palak Arora, Lorraine Croot

**Affiliations:** Barnsley Hospital NHS Foundation Trust, Barnsley, United Kingdom

## Abstract

**Case report - Introduction:**

Chikungunya is a tropical arbovirus transmitted by female Aedes Aegypti or Aedes Abopitus mosquitos. It is not indigenous to UK but occurs in epidemics in Africa and Asia. It often presents with pyrexia, arthralgia or arthritis, myalgia and a maculopapular rash and can mimic both peripheral and axial inflammatory arthritis as well as more common forms of viral arthritis. It can also become chronic leading to disabling symptoms. The diagnosis should be considered in all patients presenting with early inflammatory arthritis who have travelled to affected areas.

**Case report - Case description:**

A 57-year-old female developed sudden onset fever along with a macular rash whilst visiting South East Asia. She then developed widespread joint pains and severe inactivity stiffness, particularly affecting her ankles. The rash and fever settled after a few days, but her arthralgia persisted in her cervical spine and both small and large joints. She had a history of recurrent episcleritis and had been investigated for axial spondyloarthropathy two years previously, but MRI imaging of the spine and sacroiliac joints did not show any inflammatory changes.

Examination in the rheumatology clinic confirmed right medial epicondylitis, bilateral shoulder tenderness, tenderness over the extensor tendons of the feet and painful cervical spine movement.

Investigations revealed high inflammatory markers; CRP 29 (0-10 mg/L) and ESR 48 (0-15 mm/hr), a positive rheumatoid factor but negative anti CCP antibodies and a normal white cell count.

Acute seronegative spondyloarthropathy was suspected but Chikungunya serology was requested at the suggestion of the patient, because of the history of a mosquito bite. IgM and IgG antibodies were positive on immunofluorescence, confirming recent infection.

She was initially given intramuscular depomedrone and non-steroidal anti-inflammatory drugs (NSAIDs) with a short response but required oral prednisolone 20mg daily to suppress the inflammation in her feet. An MRI confirmed an ankle effusion and peroneal tenosynovitis. After 6 months her symptoms improved, and she was able to stop prednisolone completely and she remains well 9 months after the initial infection.

**Case report - Discussion:**

Chikungunya infection causes musculoskeletal symptoms in all affected patients, but the clinical presentation can highly variable, from mild joint pain to erosive arthritis.

It can be divided into three phases: incubation phase, acute phase, and chronic phase. The incubation phase varies between one to twelve days after the mosquito bite.

The acute phase begins with high fever, headache, polyarthralgia/arthritis, lymphadenopathy, and anorexia. Joint involvement is often distal and symmetrical affecting the hands, wrists, shoulders, knees, ankles, and feet. A maculopapular rash is common. Dengue virus and Zika virus infection can present similarly.

Treatment for acute Chikungunya fever is supportive. Analgesic, anti-pyretic and NSAIDs are used for symptom relief.

During the chronic phase, infected people develop symmetrical, migratory, oligoarticular or polyarticular arthritis with morning stiffness and joint oedema, which can last from months to years.

Our patient had a previous history which was consistent with seronegative spondyloarthropathy, an acute presentation of inflammatory arthritis and results and imaging which supported this diagnosis. The correct diagnosis could easily have been missed if a travel history had not been taken and the patient’s suspicions ignored.

The best treatment for chronic Chikungunya arthritis is unclear. NSAIDs are often the first treatment but, as in this case systemic steroids are often necessary. Conventional synthetic DMARDs have also been reported efficacious. Biologic DMARDS have been used in resistant cases.

**Case report - Key learning points:**

Chikungunya has emerged as a global disease affecting millions of people with significant musculoskeletal morbidity. Any patient has travelled to endemic areas including Africa and Asia, with fever and joint pain should be screened for Chikungunya virus as well as Dengue virus, and Zika virus.

Diagnosis is either by RT PCR (positive 0-7 days of infection or Immunoglobulin M (detectable after 5 – 10 day of infection and persists for few months).

Treatment is supportive in acute phase, may require low doses of steroids to aid resolution of symptoms. Conventional DMARDS have shown benefit in chronic phase with ongoing synovitis/tenosynovitis.

Patients may know more about rare, endemic diseases than their European doctors and their suspicions about potential diagnoses should always be considered.

